# Supplementary material for: Guidelines for Evaluating the Comparability of Down-Sampled GWAS Summary Statistics
Source: Behav Genet. 2023 Sep 15;53(5-6):404–15. doi: 10.1007/s10519-023-10152-z (PMC10584908; doi:10.1007/s10519-023-10152-z)
Supplement: Supplementary file 1 — Supplementary file1 (DOCX 2503 KB) [file 10519_2023_10152_MOESM1_ESM.docx]

Supplementary Figures

# Guidelines for Evaluating the Comparability of Down-sampled GWAS Summary Statistics

Correspondence to: [williams.m.camille@gmail.com](mailto:williams.m.camille@gmail.com), [danielle.m.dick@rutgers.edu](mailto:danielle.m.dick@rutgers.edu), [r.karlsson.linner@law.leidenuniv.nl](mailto:r.karlsson.linner@law.leidenuniv.nl)

| **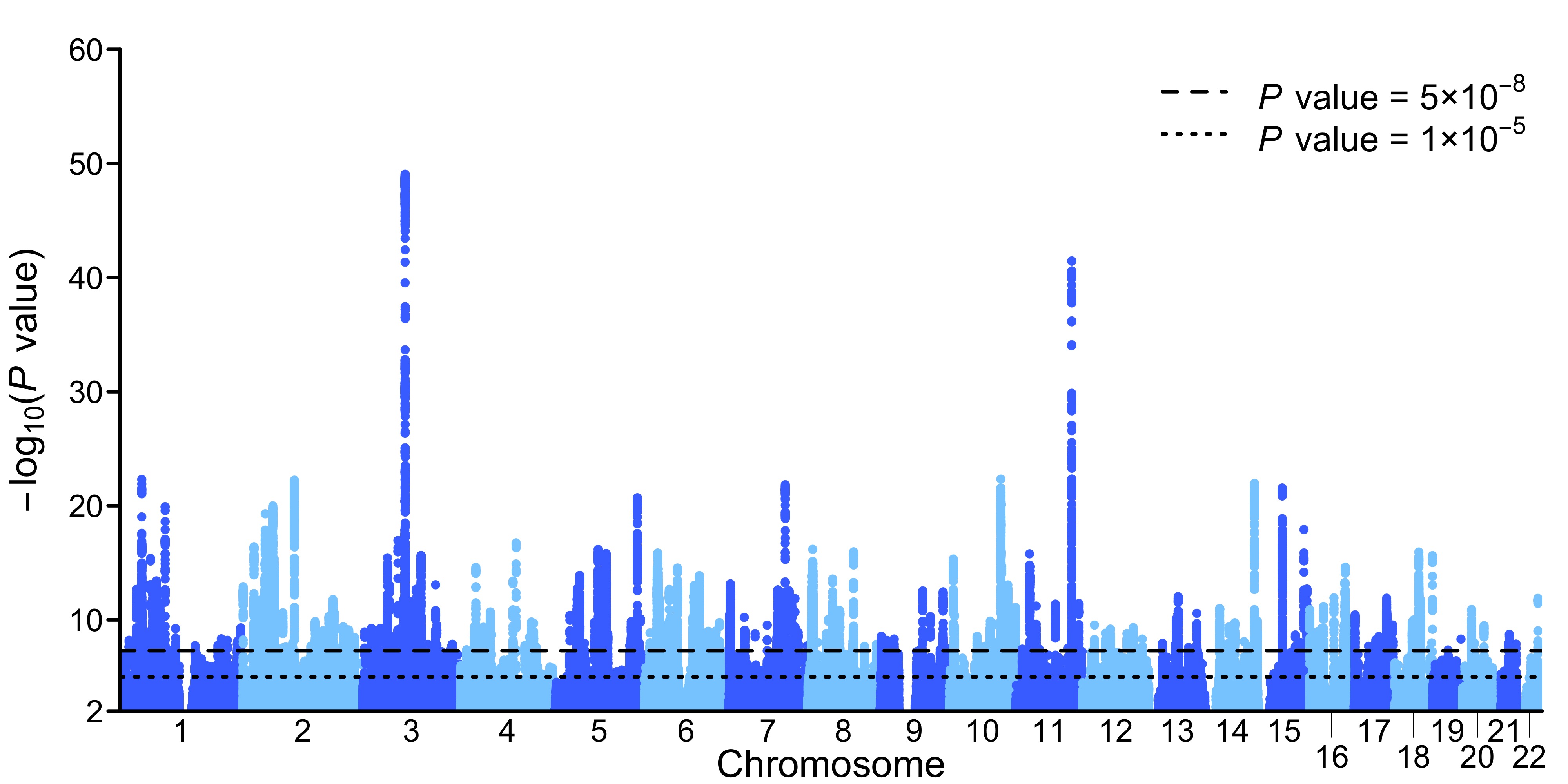** |
| --- |
| **Figure S1.** Manhattan plot of the GWAS of the EXT-min-23andMe factor, estimated with Genomic SEM (*EffN* = 1,045,957). The figure displays association *P* values on the –log10 scale (two-sided) for 505,254 with *P* < 0.01 out of 6,170,304 SNPs tested for association. The dashed line represents genome-wide significance (*P* < 5×10^–8^) and the dotted line shows suggestive significance (*P* < 1×10^–5^). A Manhattan plot of the corresponding GWAS analysis in the original study of EXT is available here: <https://www.nature.com/articles/s41593-021-00908-3/figures/2> |
| **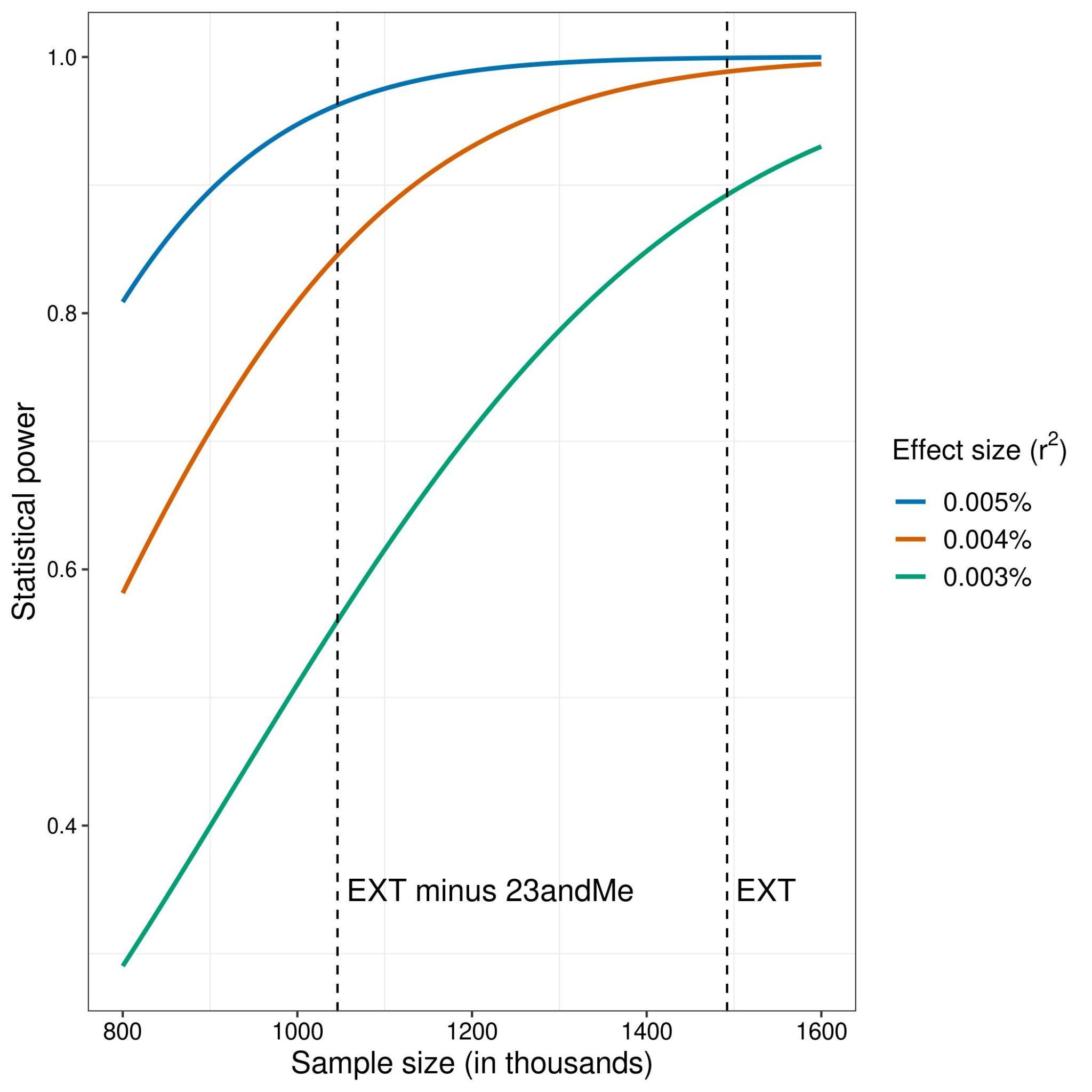** |
| **Figure S2.** Power analysis of down-sampled GWAS summary statistics. The figure displays the statistical power to detect three different effect-size magnitudes at genome-wide significance (*P* < 5×10^–8^) as a function of sample size. The three magnitudes were selected to represent smaller magnitudes that reach genome-wide significance in recent large-scale GWAS. The dashed lines mark the sample size of the original multivariate GWAS of EXT (*EffN* = 1,492,085), and that of the down-sampled multivariate GWAS of EXT-min-23andMe (*EffN* = 1,045,957). |

**
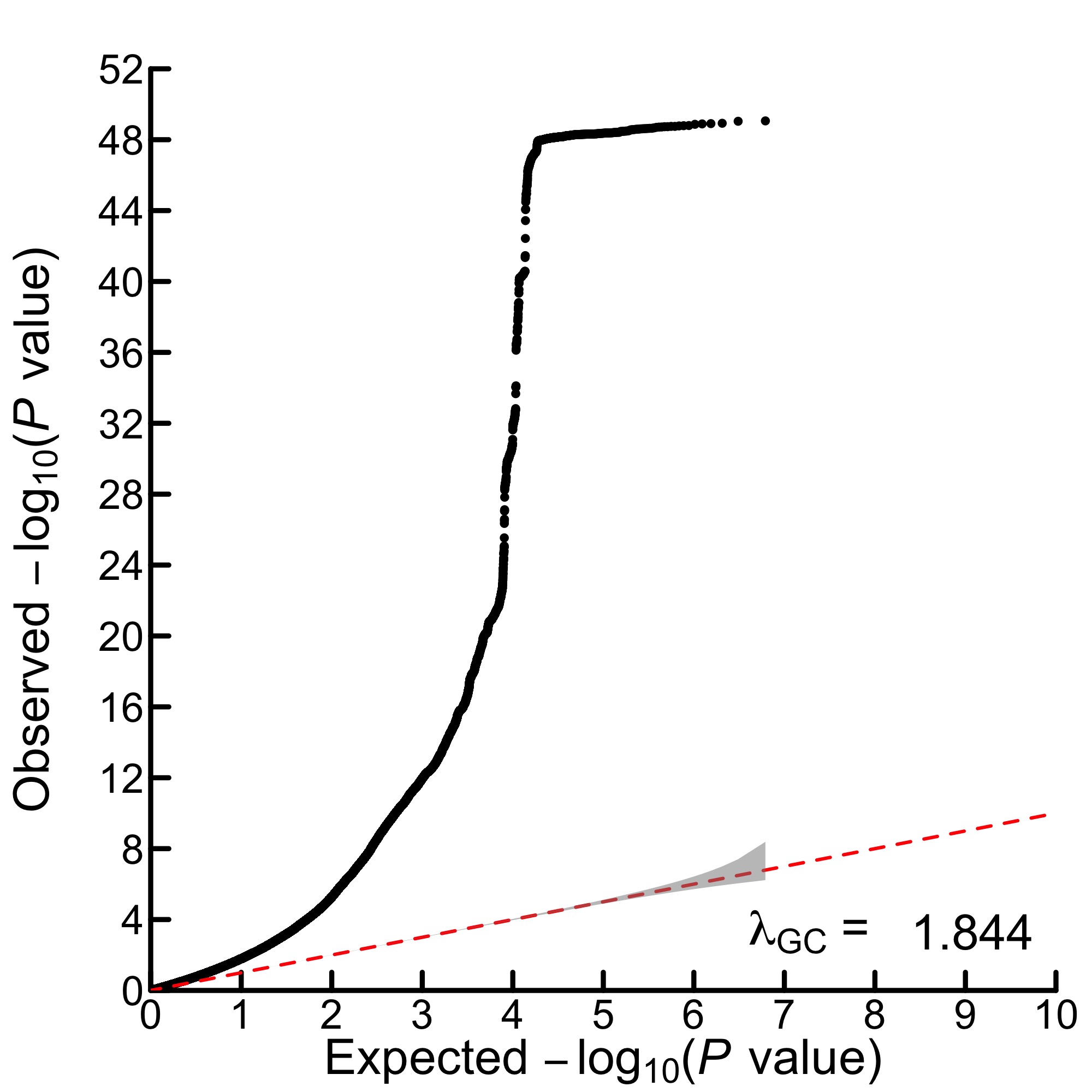
**

**Figure S3.** Quantile-quantile (Q-Q) plot. The y-axis shows the observed association *P* value on the –log10 scale (for a two-sided *Z*-test) among the 6,170,305 SNPs in the multivariate GWAS of EXT-min-23andMe (*EffN* = 1,045,957**)**, which are plotted against the expected –log10(*P*) of the null distribution. The gray shaded area shows 95% confidence intervals centered on the null distribution. The genomic inflation factor in the figure, $\lambda_{GC}$, is the median $\chi^{2}$ association test statistic divided by the expected median of the $\chi^{2}$ distribution with 1 degree of freedom. This estimate of $\lambda_{GC}$ differs somewhat from that of LD Score regression, which estimates this statistic using only ~1 million SNPs. The same plot for the original study is available here: <https://www.nature.com/articles/s41593-021-00908-3/figures/6>

| **a** | 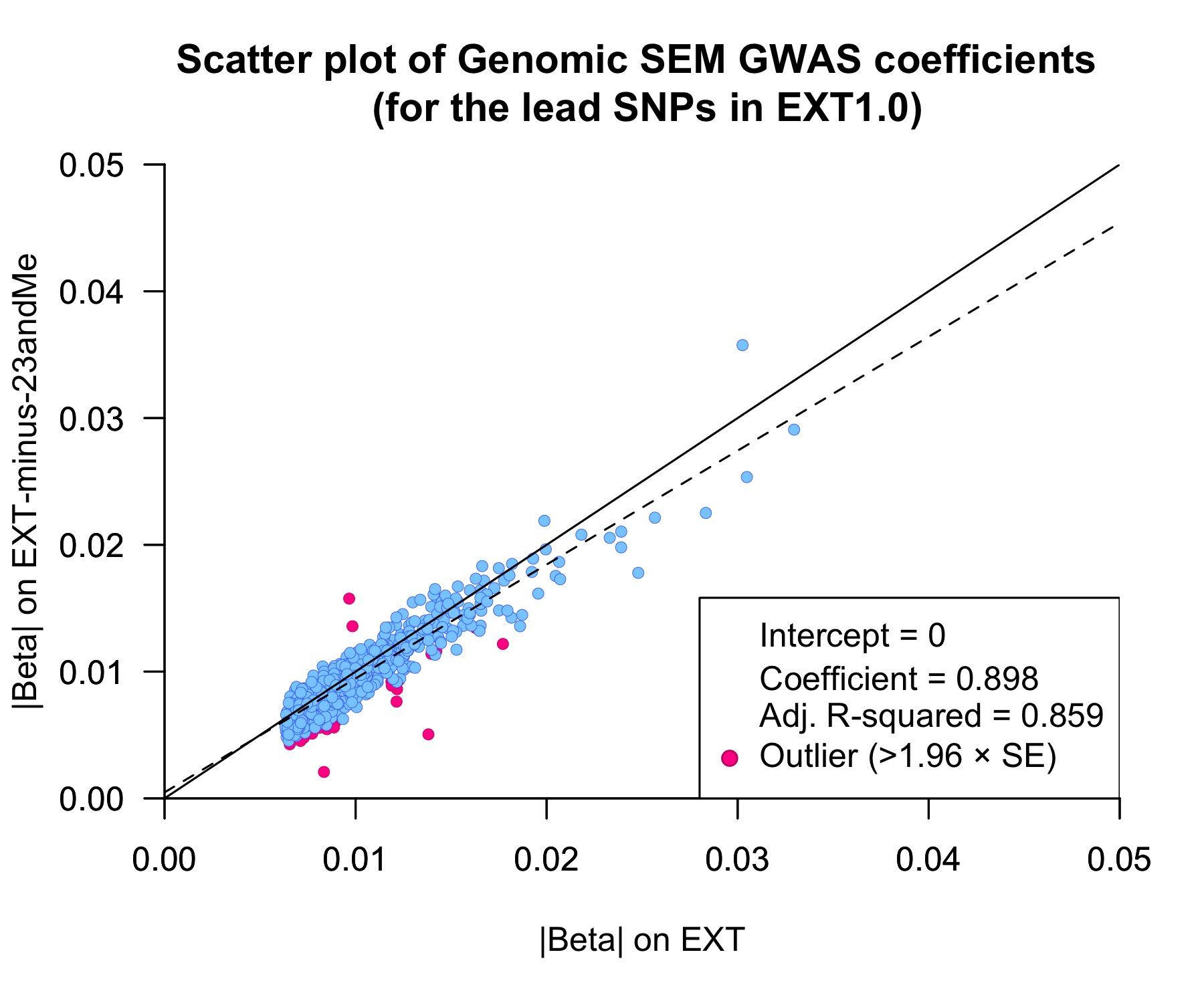 | **b** | 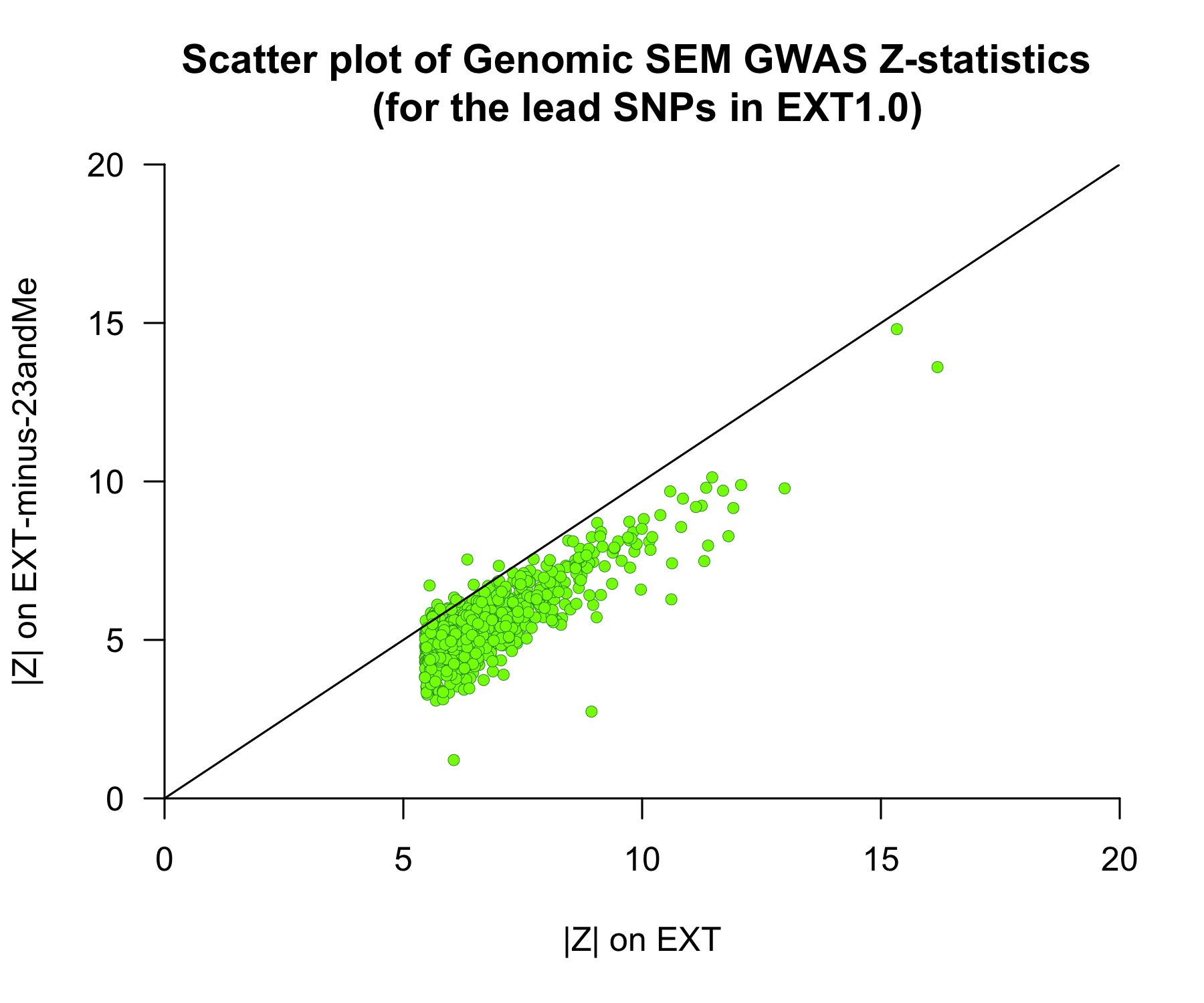 |
| --- | --- | --- | --- |
| **c** | 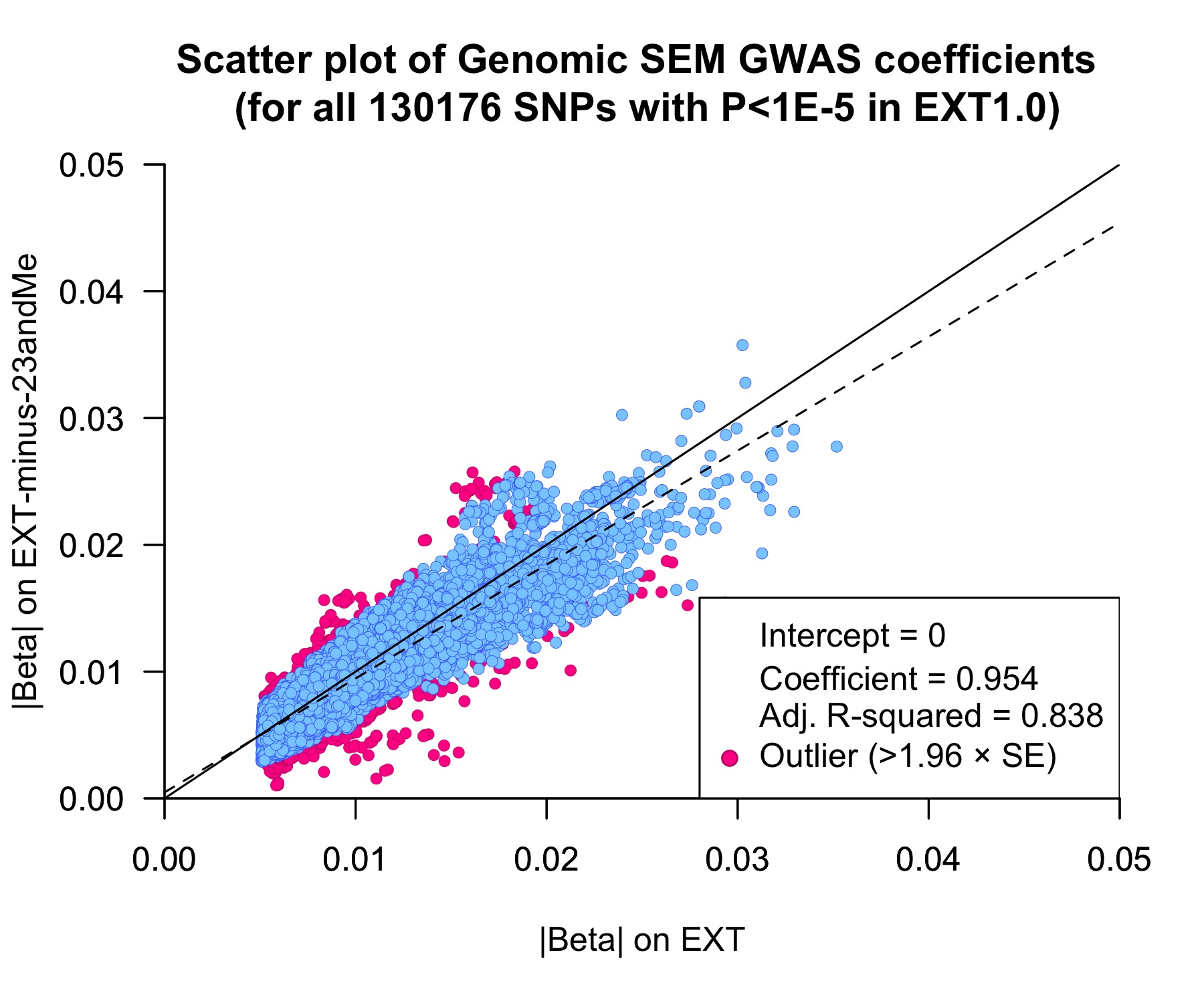 | **d** | 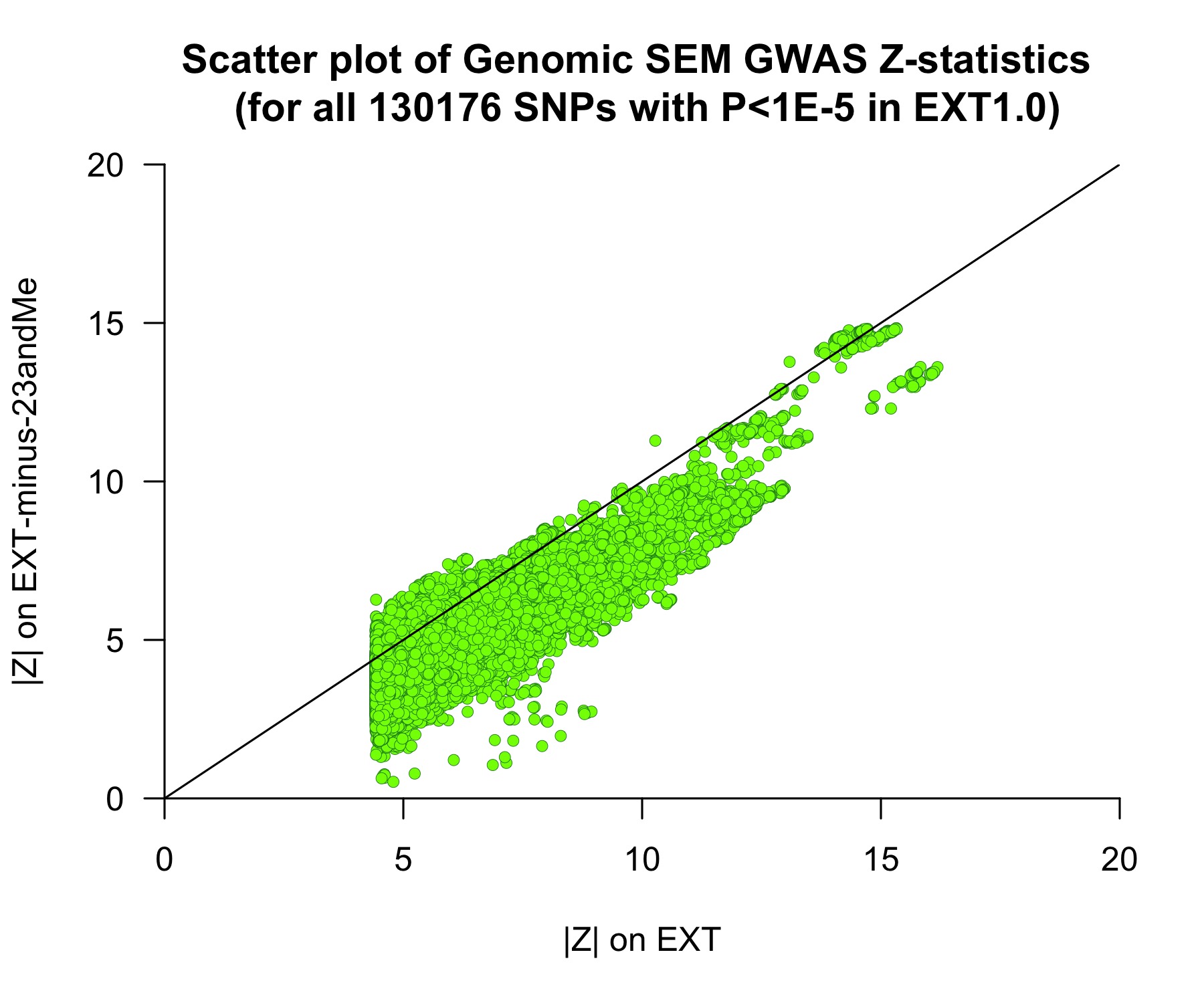 |

**Figure S4.** Scatter plots of multivariate Genomic SEM GWAS coefficients and Z-statistics for subsets of SNPs. The left panel displays the absolute value of the GWAS coefficients (“beta”) of the original multivariate GWAS on externalizing (EXT) against their estimates from the analogous down-sampled analysis (EXT-minus-23andMe), with corresponding Z-statistics in the right panel. Because the Z-statistic is a function of both sample size and the size of the GWAS coefficient, general attenuation of the Z-statistic is to be expected when down-sampling. This attenuation is noticeable by visual inspection of the scatter plots: the green dots for the Z-statistics fall systematically below the diagonal line, while the blue dots for the coefficients (for which we expect little to no attenuation) are dispersed around the diagonal, suggesting concordance in coefficients. In panels **a** and **c** there are a total of 21 (out of 842) and 2,202 (out of 130,176) outliers for which the GWAS coefficient fell outside the 95% confidence interval of the full-data estimate. Panels **a** and **c** report the results of a linear regression analysis of the observations in the figure. A diagonal solid line is plotted for reference. Dashed lines report fitted regression lines.

| 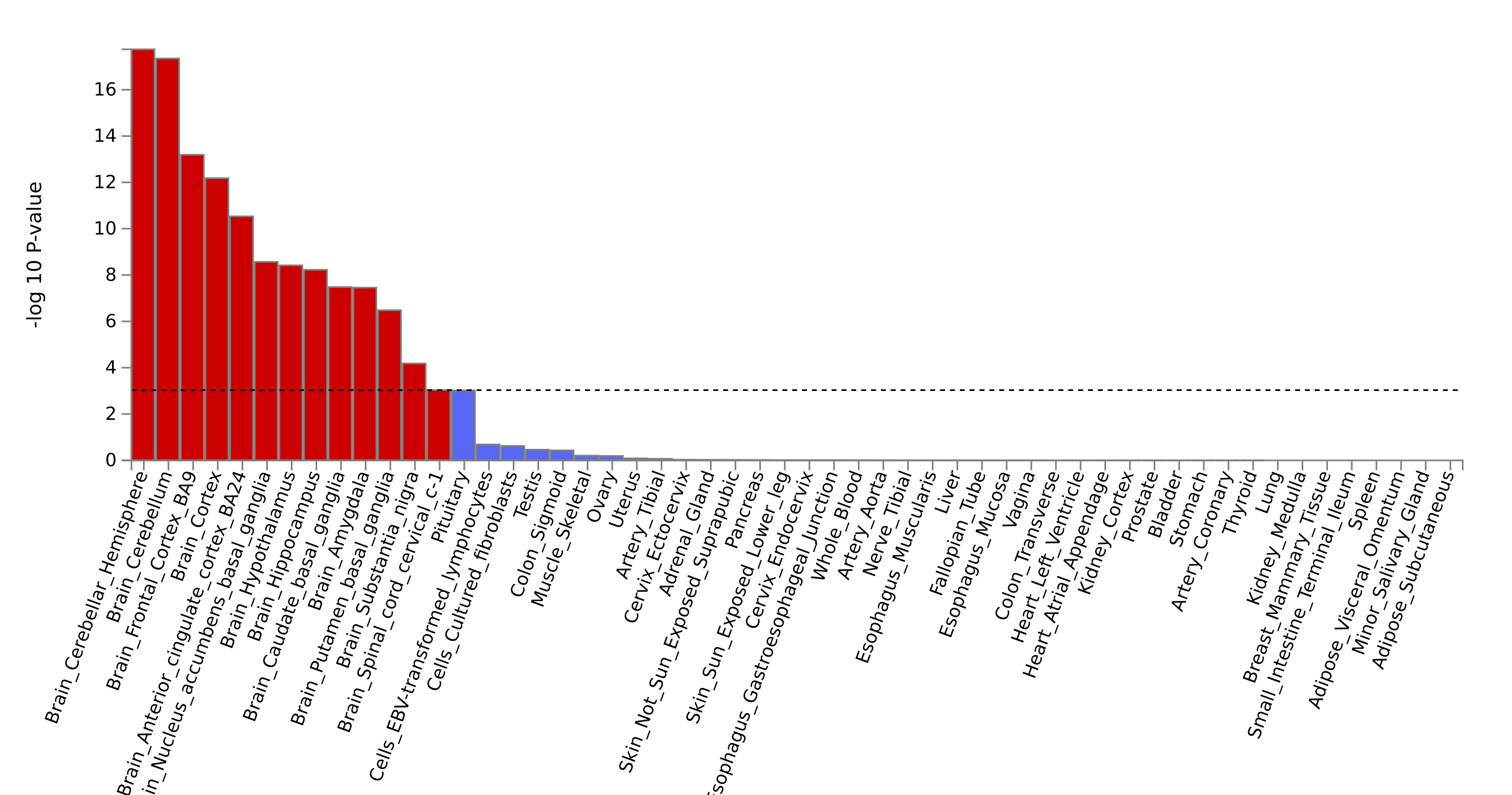 |
| --- |
| **Figures S5.** Bar plot of MAGMA gene-property analysis of enrichment in 54 bodily tissues. The figure displays *P* values on the –log10 scale (from one-sided *Z*-tests) of the point estimate from a generalized least squares regression, estimated with MAGMA as implemented in FUMA. The analysis was applied to the summary statistics from the down-sampled multivariate GWAS of EXT-min-23andMe (*EffN* = 1,045,957). Dashed line denotes Bonferroni-corrected significance, adjusted for testing 54 tissues (one-sided *P* < 9.26×10^–4^). These results are also reported in **ST3**. The same 14 tissues identified in the original study were also found significantly associated with the down-sampled multivariate GWAS of EXT-min-23andMe. The same plot for the original study is available here: <https://www.nature.com/articles/s41593-021-00908-3/figures/9> |

| 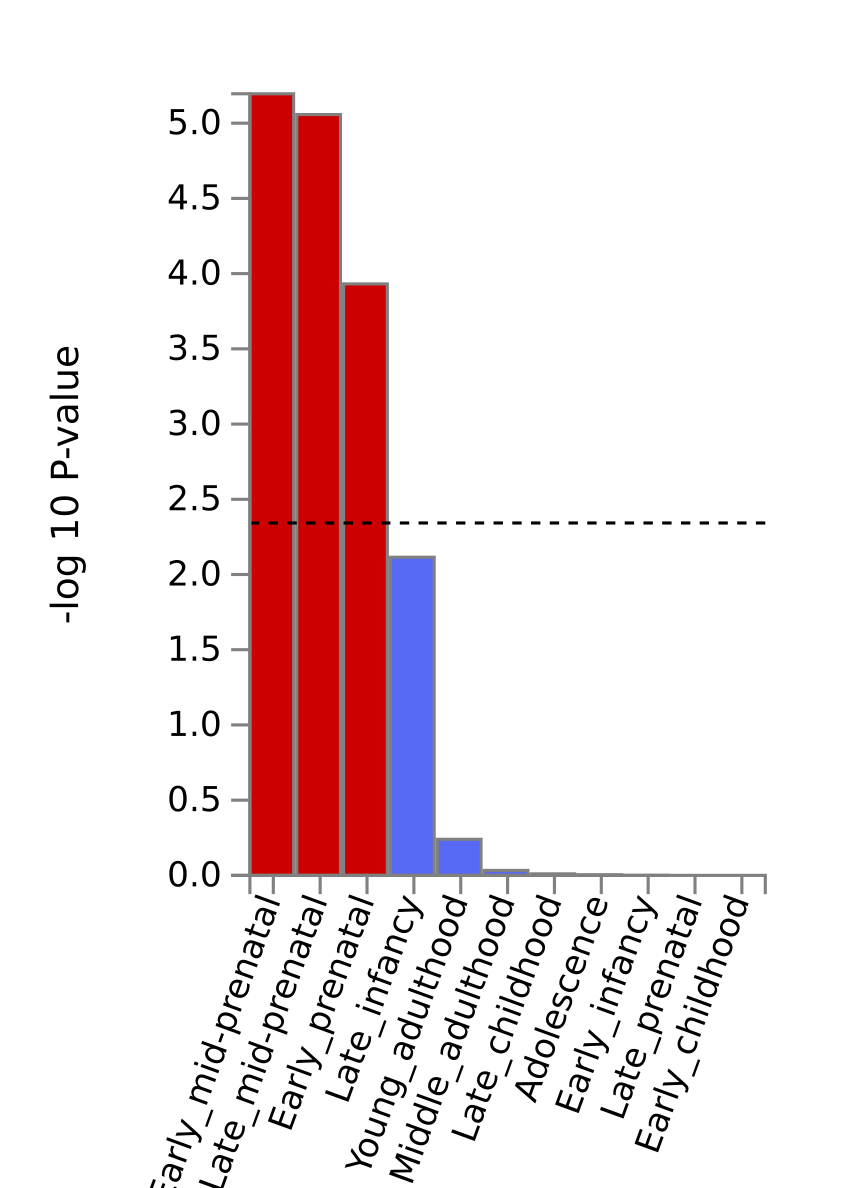 |
| --- |
| **Figure S6.** Bar plot of MAGMA gene-property analysis of enrichment in brain tissues across 11 developmental stages (BrainSpan). The figure displays *P* values on the –log10 scale (from one-sided *Z*-tests) of the point estimate from a generalized least squares regression, estimated with MAGMA as implemented in FUMA. The analysis was applied to the summary statistics from the down-sampled multivariate GWAS of EXT-min-23andMe. These results are also reported in **ST4**. Dashed line denotes Bonferroni-corrected significance, adjusted for testing 11 developmental stages (one-sided *P* < 4.55×10^–3^). The same three developmental stages identified in the original study were also found significantly associated with the down-sampled multivariate GWAS of EXT-min-23andMe. The same plot for the original study is available here: <https://www.nature.com/articles/s41593-021-00908-3/figures/10> |
